# Sustainable ultrasonic extraction of antibacterial *Basella alba* fruit dye for cotton, silk, and leather

**DOI:** 10.1016/j.ultsonch.2024.107069

**Published:** 2024-09-15

**Authors:** Loganathan Lingeshwaran, Jagadeesh Kumar Alagarasan, Seema siddharthan, Kanagasabapathy Sivasubramanian, Palanivel Velmurugan, Fatimah Oleyan Al-Otibi, Sivakumar Manickam, Moonyong Lee

**Affiliations:** a Centre for Materials Engineering and Regenerative Medicine, Bharath Institute of Higher Education and Research, Selaiyur, Chennai, Tamil Nadu 600126, India; b School of Chemical Engineering, Yeungnam University, Gyeongsan-si 38541, South Korea; c Department of Biotechnology, Saveetha School of Engineering, Saveetha Institute of Medical and Technical Sciences, Chennai 602 105, India; d Department of Biotechnology, Dhanalakshmi Srinivasan College of Arts and Science for Women, Perambalur, Tamil Nadu 621212, India; e Department of Botany and Microbiology, College of Science, King Saud University, P.O. Box 22452, Riyadh 11451, Saudi Arabia; f Petroleum and Chemical Engineering, Faculty of Engineering, Universiti Teknologi Brunei, Bandar Seri Begawan BE1410, Brunei Darussalam

**Keywords:** Ultrasound, Extraction, Red dye, *Basella alba*, Dyeing, Color fastness

## Abstract

Alternative to synthetic dyes containing harmful compounds, dyes derived from natural sources are gaining popularity due to their safer and eco-friendly nature. This study focuses on extracting red dye from *Basella alba* fruit and optimising the extraction methods, including ultrasonic bath, ultrasonic probe, and direct heating. The extracted dye was then used for dyeing cotton, silk, and leather without needing a mordant. Furthermore, the antibacterial properties of the extracted red dye were evaluated against skin bacteria. The UV–Visible spectrophotometric analysis revealed that the maximum red colour in the methanol extract (λ_max_ 270 and λ_max_ 542 nm) was achieved at 60 °C for 30 min using the ultrasonic water bath extraction method, followed by the ultrasonic probe and direct heating methods. The FTIR spectra confirmed the presence of flavonoids, betacyanin, and gomphrenin-I in the extracted dye. The ultrasonic dyeing process at 50 °C yielded a K/s value 6.3 for the dyed cotton, silk, and leather without using a mordant. Additionally, the fatness test indicated a high grade of 0.5–1.5 for the ultrasonic dyeing method compared to other dyeing techniques. The extracted dye exhibited significant antibacterial activity against all *Pseudomonas* sp. after extraction in methanol, with the highest inhibition observed against *Pseudomonas* sp. with a MIC of 1.56 mg/ml.

## Graphical abstract

**Figure:**
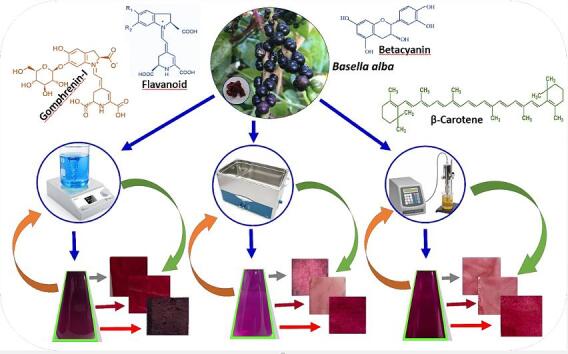

## 1 Introduction

Sustainable green technology is steadily becoming more prevalent in the textile industry. Alternatively, textile sectors use synthetic dyes due to their high colour stability and low cost, which leads to environmental issues associated with textile effluents [1], [2]. A fundamental problem with textile effluents is that they contain substantial amounts of chemicals (acids, bases, and salts) as well as various dyestuffs (direct, reactive, dispersion, and cationic dyes, for example) [3]. Contaminants such as these seriously threaten the ecosystem, putting the lives and health of humans and animals at risk [4]. As a result, scientists are increasingly involved in replacing synthetic chemicals with eco-friendly alternatives without sacrificing output quality or efficiency. The dyes used in textiles before 1856 were derived primarily from natural sources, which have a long history of use [5]. Global environmental awareness and the energy crisis have increased interest in studying natural dyes, even if synthetic dyes eventually replace natural dyes [6]. Natural dyes have several advantages over synthetic dyes, including their non-toxic, noncarcinogenic, and biodegradable nature [7]. Natural dyes may also be obtained from renewable natural resources such as plants, flowers, seeds, insects, bark, roots, and microorganisms [8]. Environmental protection laws have been enacted worldwide to ensure ecological safety, environmental preservation, and sustainable growth in the textile industry [9]. The textile industry prioritises safety, sustainability, and wastewater treatment to reduce pollution [10]. The cost of textile wastewater treatment systems is unaffordable for some companies. Textile waste can be reduced through eco-dying and biofinishing without costly and inefficient effluent treatment [11]. In order to produce eco-textiles sustainably and in a cleaner way, researchers worldwide are developing green technologies and sustainable dyes.

Using high-frequency sound waves, natural dyes are extracted using an ultrasonic technique that releases the pigments or dyes from organic materials. The main concept behind this approach is to immerse plant components or other sources of dyes in a solvent (such as water or ethanol). When ultrasonic waves are applied to the combination, they create cavitation, or the development and bursting of small bubbles, which enables the dyes to be released into the solvent more efficiently [12]. Optimizing extraction efficiency requires operating at regulated temperatures. Compared to traditional procedures, ultrasonic extraction yields more natural colors, uses less solvent, and completes the extraction process faster. Furthermore, because it frequently calls for lower temperatures and shorter extraction periods, it is seen as a greener process because it uses less energy and has a less environmental impact [13].

A member of the basellaceae family, Malabar spinach (*Basella alba*) is characterised by its ascending, branching stems covered in succulent and mucilaginous leaves. The fruits of Malabar spinach contain betalains, including gomphrenin I (betanidin 6-O-D-glucoside) and its isoform, as well as carbohydrates, proteins, lipids, niacin, ascorbic acid, and tocopherols [14]. The betalains are pigmented nitrogen compounds that are water-soluble. Amanita and Hygrocybe fungi and most Caryophyllales plants possess these fungi [15]. The chromophore core consists of balsamic acid, which condenses with *cyclo*-DOPA, amino acids, or amines to produce red-violet betacyanins and yellow-orange betaxanthins. Both gomphrenins and betacyanins contain glucose at C-6 and a hydroxyl group at C-5. Betacyanins are accompanied by isobetacyanins in plant extracts [16].

Cotton fibres in the Gossypium genus are among the most widely recognised natural fibres. They consist of cellulose and 1,4-d-glucopyranose structural units [17]. On the other hand, silk is a luscious and durable material derived from proteins. Silk introduces diversity and richness to natural dyes [18]. Fresh hides primarily contain water, proteins, fatty substances, and mineral salts. In the production of leather, the focus lies on protein utilisation. Collagen derived from tanning is the primary protein source for leather production, while keratin is from hair, wool, horn, and epidermal tissue [19]. Natural dyes often exhibit poor lightfastness and limited colour absorption, emphasising the need for improved harvesting methods for plant-based dyes specifically tailored for silk dyeing [20]. Traditional solvent extraction techniques for natural dyes have proven inefficient due to the heavy reliance on organic solvents, resulting in longer extraction times, lower yields, and limited efficiency [21].

Implementing ultrasound techniques can accelerate mass transfer rates and facilitate the disruption of cell walls by generating microcavities. This, in turn, leads to higher product yields, reduced solvent consumption, and shorter processing times [22].

This study uses an environmentally friendly ultrasound-assisted extraction method to dye cotton, silk fabrics, and leather while imparting antibacterial properties. The investigation encompasses the extraction process, dye uptake, color intensity, and dyeing fastness of *B. alba* fruit dye for the materials mentioned above, utilising techniques such as an ultrasonic probe, ultrasonic water bath, and direct heating.

## 2 Materials and methods

### 2.1 Chemicals and media

Analytical-grade chemicals and solvents procured from SRL India were employed in this study. The media utilised in the experiments was obtained from Hi-media (Mumbai, India).

Tetracycline, a reference antibiotic, was utilised for comparison purposes.

### 2.2 Natural dye and dyeing materials

Fresh *Basella alba* fruits were collected from the foothills of Kolli Hills near Namakkal, Tamil Nadu, India. For dye extraction, *B. alba* fruits were carefully separated from the peduncle, and the outer surface of the fruits was thoroughly cleaned using ample water. For subsequent analysis, commercially available cotton fabric (density, 190 gm^−2^), silk fabric (density150 g/m^2^), and tanned goat leather were purchased from the local market and were cut into samples, each weighing 1 g.

### 2.3 Ultrasound (US) water bath-assisted dye extraction from *B. alba* fruit

To extract the dye, 5 g *B. alba* fruits were added to an extraction vessel containing 50 ml of various solvents, including double distilled water, ethanol, methanol, and ethanol: methanol (1:1). The vessel was covered with aluminum foil to prevent evaporation during the extraction process. Subsequently, the extraction vessel was placed in an ultrasonic water bath with a capacity of 20 L and operating at a frequency of 40 kHz and an input power of 500 W. The internal temperature of the vessel was maintained at 80 °C. The ultrasonic bath featured four transducers positioned at the bottom of a tank with internal dimensions measuring 500 × 300 × 200 mm^3^. In order to determine the optimal extraction time for achieving a higher dye extraction rate, the extraction time was incrementally increased by 10 min. Samples were collected at 10 min intervals, and their absorbance wavelengths were measured between 200 and 800 nm using a UV–Vis spectrophotometer (UV-1800, Genesys 180, Thermo Fisher Scientific, USA) up to 60 min [23].

### 2.4 Ultrasonic probe-assisted dye extraction from *B. alba* fruit

An extraction vessel was prepared by adding 5 g of *B. alba* fruits to 50 mL of various solvents, namely double distilled water, ethanol, methanol, and ethanol:methanol (1:1 ratio)). The vessel was then placed in an ultrasonic probe sonicator (LABMAN, Pro-650, India) and operated for 30 min at different input powers of 390 W, 455 W, 520 W, 585 W, and 650 W. The temperature in the vessel was maintained at 60 °C. The absorbance wavelength of the dye was measured using a UV–visible spectrophotometer for up to 60 min [24].

### 2.5 Heating mantle (direct heat) assisted dye extraction from *B. alba* fruit

An extraction vessel containing 50 ml of different solvents (double distilled water, ethanol, methanol, and ethanol:methanol (1:1)) and 5 g *B. alba* fruits were subjected to heating on a mantle at different temperatures (30 °C, 40 °C, 50 °C, 60 °C, and 70 °C) for 10 min. The absorbance wavelength of the dye was measured using a UV–visible spectrophotometer within the range of 200 to 800 nm for a maximum period of 60 min [24].

### 2.6 Optimisation of natural dye extraction from *B. alba* fruit

The extraction of natural dye from the fruit of *B. alba* involved optimising several parameters. The concentration of cherry red dye powder was varied within the range of 1–5 % to determine the optimal concentration for extraction. The extraction process was also carried out at different temperatures (30 °C to 70 °C with 10 °C intervals) and for varying durations (10 to 60 min). The intensity of the dye colour was measured using a UV–visible spectrophotometer. The selection of optimal dye extraction conditions was based on the colour intensity achieved through the extraction process [25].

### 2.7 FTIR analysis

Fourier Transform Infrared (FTIR) spectroscopy (Thermo Fisher, Summit Lite, USA) was employed within the range of 500 to 4000 cm^−1^ to identify the functional groups in the extracted dye. Specific functional groups could be identified by cataloguing and categorising the observed vibrational modes in the dye.

### 2.8 Optimisation of dyeing process parameters for cotton, silk fabric, and leather

The dyeing process for cotton, silk fabric, and leather involved the utilisation of the extracted cherry red dye employing the same extraction methods, i.e., ultrasonic water bath, ultrasonic probe, and direct heating. Before dyeing, the cotton and silk materials underwent thorough washing and bleaching procedures. Similarly, the leather surface was cleaned to remove hair follicles. A consistent dye concentration of 12 % was maintained for all dyeing trials. The dyeing materials were cut into square pieces measuring 1″× 1″. To optimise the dyeing conditions, temperature adjustments (40 °C, 50 °C, 60 °C, 70 °C, and 80 °C) were made for 30 min in an ultrasonic bath operating at 40 kHz. An ultrasonic probe was used at different power levels (390 W, 455 W, 520 W, 585 W, and 650 W) for 30 min. Conventional heating was employed at 30 °C, 40 °C, 50 °C, 60 °C, and 70 °C for 10 min to optimise the dyeing conditions. After dyeing, the samples were washed in cold water using a liquor ratio (L:R) of 50:2. A nonionic detergent (laboratory detergent) was used at a concentration of 3 g/l to remove any remaining unfixed dye, and the samples were allowed to dry naturally [26].

### 2.9 Dye uptake

The dye uptake of *B. alba* fruit dye was evaluated using a UV–Vis spectrophotometer with some minor modifications. Eq. (1) was employed to calculate the dye uptake for cotton, silk fabric, and leather.

(1) $$\mathrm{Dyeuptake}=\frac{(A0\;-At)\;}{A0\;}X100\%$$

The absorbance of the initial dye liquor is denoted as A_0_, while the absorbance of the dye liquor at different stages is represented as A_t_.

### 2.10 Color strength

The CIE (L*, a*, b*, ΔE, and K/S value) of dyed cotton, silk fabric and leather were assessed using a Datacolour 600 spectrophotometer in the scanning range of 200 to 800 nm and at a 10° observation angle. On the 0–100 scale, higher values indicate lighter shades of fabric, while the L* parameter represents the degree of lightness or darkness. The scale of green to red is also reflected, where negative values indicate green hues and positive values indicate red hues. Negative values are assigned to blue hues, while positive values are assigned to yellow hues on the blue/yellow scale (B) [27]. The test result was derived by averaging eight readings obtained from different positions. The K/S value was determined by employing Eq. (2).

(2) $$K/S=\frac{{\left(1-R\right)}^{2}\;\text{-}\;{\left(1-R_{0}\right)}^{2}\;}{2R-2R_{0}}$$

S represents the scattering coefficient, while *k* represents the absorption coefficient. R and R_0_ represent the reflectivity of dyed and undyed cotton, silk fabric, and leather, respectively. Furthermore, the CIE lab color space was utilised to describe the color appearance of the silk fabric.

### 2.11 Fastness properties

As per the study conducted by Otaviano et al. [27], the fastness of dyed samples to light, washing, perspiration, and rubbing was evaluated using the following standards: ISO 105B02:1994 (method 1, exposure cycle A1), ISO 105-C06:2010 (method A1S), ISO 105E04:2014, and ISO 105-X12:2016. The ratings for fastness range from 1 (poor) to 5 (outstanding), with intermediate ranking levels of good (4) and reasonable (3).

### 2.12 Antibacterial properties of leather and colored fabrics

Following the studies conducted by Zhu et al. [28] and Wang et al. [29], with minor modifications, the antibacterial activity of dyed cotton, silk fabrics and leather against *Staphylococcus sp, Pseudomonas* sp, *Vibrio* sp, *Klebsiella* sp, and *Micrococcus* sp (Obtained from Bharath Medical College and Hospital) was assessed following the AATCC 100–1993 test criteria. To experiment, a bacterial culture with a concentration of 1.0 × 10^5^ CFU/mL was prepared in Muller Hinton broth. Dyed cotton, silk fabrics and leather samples, along with their respective controls (2 cm × 2 cm), were placed in separate 150 ml beakers containing 50 ml of Muller Hinton broth and bacteria. The beakers were then incubated in a shaking incubator at 37 °C for 24 h with agitation set at 100 rpm. Following the incubation period, 1 mL of media from each beaker was extracted and subjected to three consecutive serial dilutions. From the 10^-3^ dilutions, the bacterial solution was streaked onto Muller Hinton agar plates. These plates were then incubated at 37 °C for 24 h to allow for the estimation of the bacterial load. A control group was included, consisting of liquid media without any samples. Following incubation, the viable bacteria were counted, and the results have been presented according to the following Eq. (3), which determines the bacteriostasis rate (R) of cotton, silk fabrics, and leather.

(3) $$R\%=\left(A-B\right)/A\times 100$$

Where A represents the number of bacterial colonies in the medium for raw wool, and B presents the number of bacterial colonies in the medium for coloured materials and leather.

### 2.13 Data analysis

The statistical analysis was performed using the Statistical Analysis System (SAS). Each sample was measured three or more times and the average of the measurements was taken as the response variable.

## 3 Results and discussion

Ultrasound has been employed for a considerable period to extract natural dyes from plant sources and colour textile fabrics in a cost-effective and environmentally beneficial manner. This approach helps minimise the utilisation of chemicals, time, energy, and effluent in the dyeing process [30]. Ultrasound-assisted extraction of *Hawthorn* fruit dye in dyeing polyamide fabric has been studied by Sadeghi-Kiakhani et al. [31]. The research also explored the biological applications of the extracted dye. Betalains, anthocyanins, chlorophylls, and carotenoids are plant-based pigments or dyes that find applications in various industries. They are utilised as food additives, textile colorants, livestock feed additives, pharmaceutical ingredients, and cosmetic components [32]. To determine the optimal dye extraction conditions and achieve maximum yield, the relative colour strength value of the extracted dye was evaluated. Different extraction methods were employed, resulting in varying levels of colour strength due to alterations in time, temperature, and solvent used during the extraction process.

**Fig. 1:**
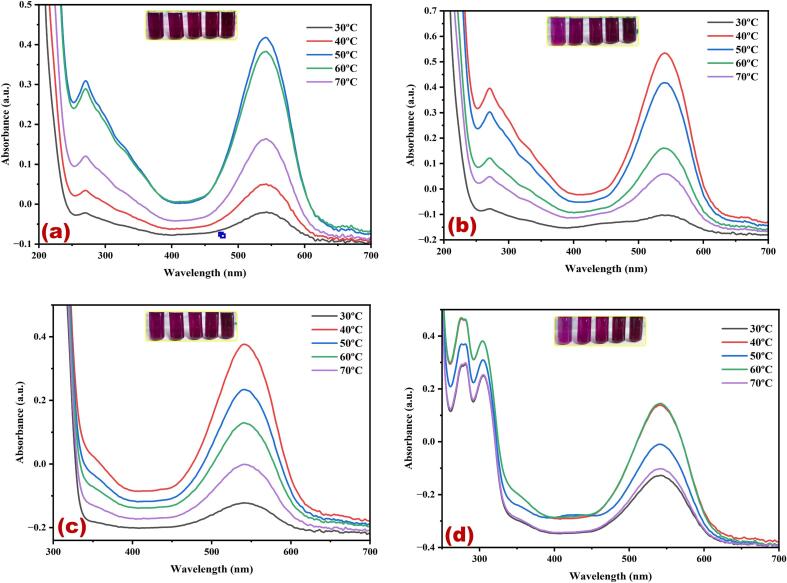
Optimization of *Basella Alba* fruit red dye extraction using direct heat in different temperatures and solvents (a) water, (b) methanol, (c) ethanol, and (d) methanol: ethanol. (For interpretation of the references to color in this figure legend, the reader is referred to the web version of this article.)

**Fig. 2:**
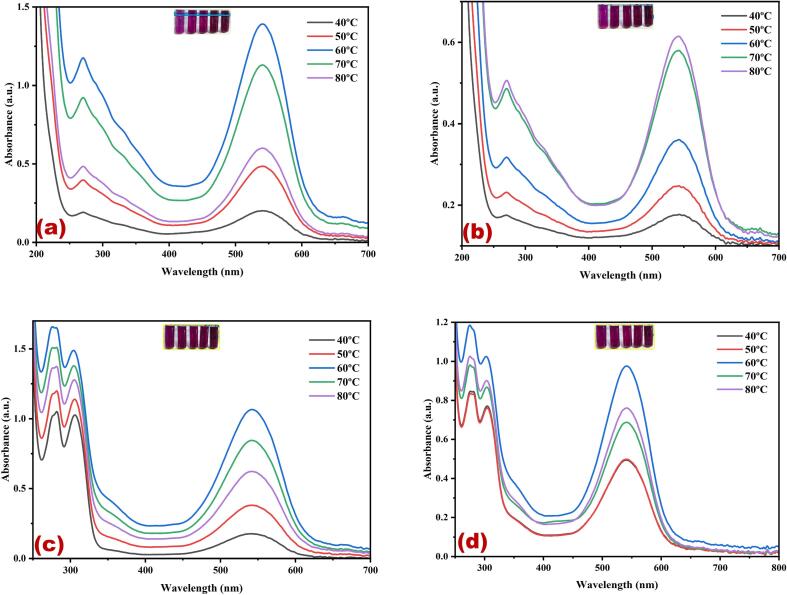
Optimization of *Basella Alba* fruit red dye extraction using an ultrasonic water bath in different temperatures and solvents (a) water, (b) methanol, (c) ethanol, and (d) methanol: ethanol. (For interpretation of the references to color in this figure legend, the reader is referred to the web version of this article.)

**Fig. 3:**
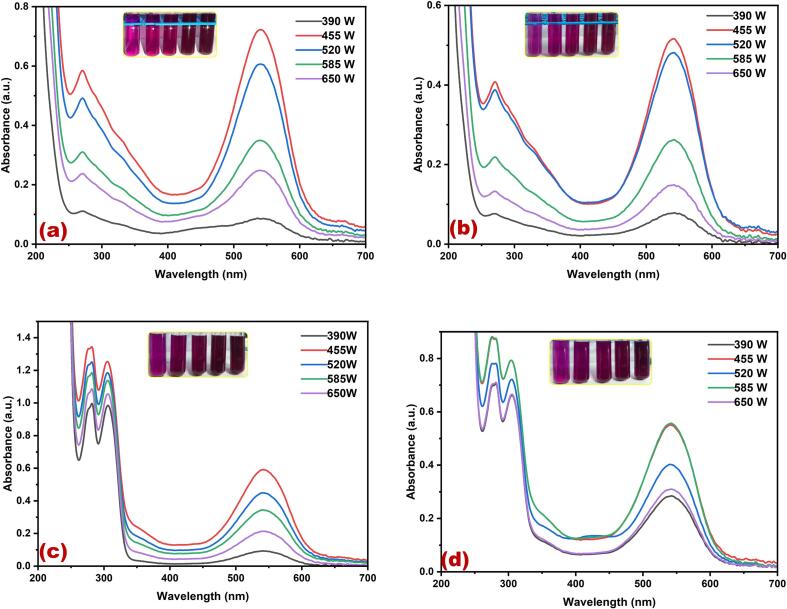
Optimization of *Basella Alba* fruit red dye extraction using an ultrasonic probe in different output power (W) and solvents (a) water, (b) methanol, (c) ethanol, and (d) methanol: ethanol. (For interpretation of the references to color in this figure legend, the reader is referred to the web version of this article.)

Fig. 1(a-d) presents the UV–Vis spectral analysis results, indicating the maximum yield of cherry red color dye obtained in different solvents using ultrasonic water bath extraction (Fig. 1a water, Fig. 1b methanol, Fig. 1c ethanol, and Fig. 1d methanol: ethanol (1:1)). In the ultrasonic water bath extraction method, the optimum dye yield was achieved under the following conditions: 50 °C, 40 °C, and 60 °C for 30 min in water, methanol, ethanol and ethanol methanol mixture, using 5 g of fruit in methanol, respectively. The results showed that 60 °C provided the highest extraction yield without noticeable degradation, making it the ideal choice for this study. he extraction efficiency increased up to 30 min, after which the yield plateaued, indicating that 30 min was sufficient to maximize dye extraction. Prolonging the extraction time beyond 30 min did not result in a significant increase in yield and posed a risk of potential degradation of the extracted compounds. The relative colour strength values gradually decreased at lower temperatures and subsequently increased with both temperature and duration of the extraction process. The cavitation effect induced by ultrasound leads to the rupture of cell walls, allowing the release of their contents into the surrounding medium. This effect is further enhanced by the mechanical effects of ultrasound, facilitating greater solvent penetration into the fruit cells [33]. Consequently, this method offers the advantage of generating higher yields faster and at lower processing temperatures. Moreover, various applications have shown that ultrasound-assisted extraction is an environmentally and economically viable alternative to traditional methods for extracting natural resources. The key advantages of ultrasound-assisted extraction include reduced extraction and processing times, decreased energy and solvent consumption, minimised unit operations, and lower CO_2_ emissions [33]. These benefits contribute to more efficient and sustainable extraction processes. The cherry red dye displayed two peaks in its spectrum at λ_max_ 270 nm and λ_max_ 542 nm. These peaks are likely associated with flavanoids and betacyanins, well-known pigment compounds in various plant sources. Gomphrenin-I, belonging to the Betalain family, is a prominent red pigment in dye extracts. It is a significant constituent of betacyanins [34]. *Basella alba,* commonly known as Malabar spinach or vine spinach, contains a significant amount of β-carotene. As the fruit ripeness, the quality of gomphrenin-I also improves. In ripe fruits, the concentration of gomphrenin-I reaches 36.1 mg per 100 g of fresh weight [34]. Fig. 2(a-d) shows the UV–visible spectrum of the cherry red dye extracted from *B. alba* fruit using different solvents in an ultrasonic water bath. The extraction process involved different temperatures and solvents: 60 °C (Fig. 2a) for water, 80 °C (Fig. 2b) for methanol, 60 °C (Fig. 2c) for ethanol, and 60 °C (Fig. 2d) for a methanol-ethanol mixture. The extraction duration for all solvents was 30 min. The uniform ultrasound produced in hot water using an ultrasonic bath facilitates the gradual release of the dye into the solvent system. Hence, the stability of the red dye remains unchanged throughout the extraction process. Three different polar solvents and a combination of solvents were used to extract the red dye. Among the three extraction methods, methanol alone exhibits a superior dye yield compared to other solvents. This can be attributed to the strong interaction between methanol and dye molecules, which enhances the extraction efficiency. Fig. 3(a-d) shows that the highest dye yield can be achieved by employing the ultrasonic probe extraction method at 50 °C for 30 min, using 5 g of the flower. Among the various solvents and kHz used for extraction, the use of 455 W power enhances dye extraction for all the solvents: water (Fig. 3a), methanol (Fig. 3b), ethanol (Fig. 3c) and methanol-ethanol mixture (Fig. 3d) for 30 min. Moreover, for the methanol-ethanol mixture, 585 W power further enhances dye extraction (Fig. 3d).

**Fig. 4:**
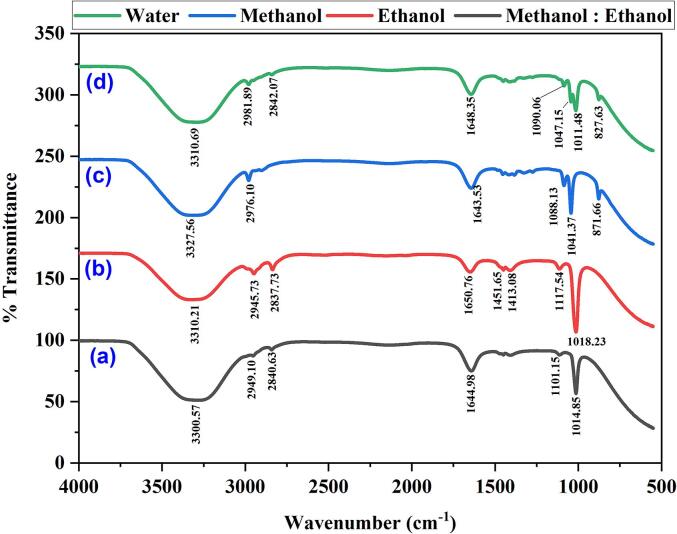
FT-IR spectra of optimized *Basella Alba* fruit red dye extraction using an ultrasonic water bath and various solvents (a) Water, (b) methanol, (c) ethanol, and (d) methanol: ethanol. (For interpretation of the references to color in this figure legend, the reader is referred to the web version of this article.)

Fig. 4(a-d) presents the FTIR spectra in the spectral range of 4000 ∼ 400 cm^−1^, depicting the optimal *B. alba* red dye yield achieved using an ultrasonic water bath in a methanol and ethanol mixture. Fig. 4a shows the spectrum of the red dye obtained by ultrasonically dissolving it in water. The observed peaks at 3300 cm^−1^, 2949 cm^−1^, 2840 cm^−1^, 1644 cm^−1^, 1101 cm^−1^, and 10148 cm^−1^ correspond to O-H stretching vibration, asymmetric and symmetric stretching of alkanes groups (H-C-H), stretching vibration of C=C bonds, carbonyl (C=O) group stretching, and stretching of C-O bonds, respectively. In Fig. 4b, the absorption band observed at 3310 cm^−1^ is attributed to the O-H stretching of alcohol and water. The peak at 2945 cm^−1^ corresponds to −C-H stretching in aldehydic, the peak at 2837 cm^−1^ belongs to –CH stretching, the peak at 1650 cm^−1^ belongs to C-F stretching, and the peak at 1018 cm^−1^ is attributed to C-O starching. The peaks observed at 871 cm^−1^ and 1088 cm^−1^ can be assigned to the aromatic C–H bending vibration and the C-O-C bond of polyphenols, respectively (Fig. 4c). The other remaining peaks in the spectrum are similar to those observed in the methanol extract (Fig. 4b). In Fig. 4d, the peaks observed between 3310 cm^−1^and 400 cm^−1^ for the methanol and ethanol extract exhibit similarities to those observed in the methanol and ethanol spectra alone. The characteristic peaks resemble those reported for anthraquinone-containing compounds in previous studies [35], [36], [37], [38]. Flavonoids are defined by hydroxyl (–OH) and carbonyl (C=O) functional groups. FTIR peaks corresponding to flavonoids are associated with hydroxyl group stretching vibrations (–OH) near 3400–3200 cm^−1^. The presence of phenolic compounds indicates flavonoids. Flavonoids’ carbonyl (C=O) group produces stretching vibrations, resulting in a strong peak between 650 and 1600 cm^−1^. Peaks in the 1500–1450 cm^−1^ range correspond to flavonoid aromatic rings’ C=C stretching vibrations. Betacyanins, nitrogen-containing dyes, have distinctive FTIR peaks associated to functional groups including hydroxyl, carboxyl, and amine groups. Betacyanin structures have a peak between 3400 and 3300 cm^−1^, indicating stretching vibrations of hydroxyl (–OH) groups. Betacyanin molecules exhibit peaks at 1720–1700 cm^−1^ due to carboxyl (C=O) group stretching vibrations. Betacyanin conjugated systems have a C=N stretching vibration peak at 1630–1600 cm^−1^. In spite of its conjugated structure and hydroxyl and carboxyl groups, gomphrenin-I betacyanin shows distinct peaks. Hydroxyl groups in gomphrenin-I encounter O-H stretching vibrations, as shown by the elevated peak at 3300–3200 cm^−1^. GomphreninI has a significant peak between 1710 and 1680 cm^−1^, indicating stretching vibrations of the carboxyl group (C=O). The peak in the 1600–1550 cm^−1^ region is connected to the stretching vibrations of the C=N group, which indicate the chromophore structure of betacyanin derivatives like gomphrenin-I.

**Fig. 5:**
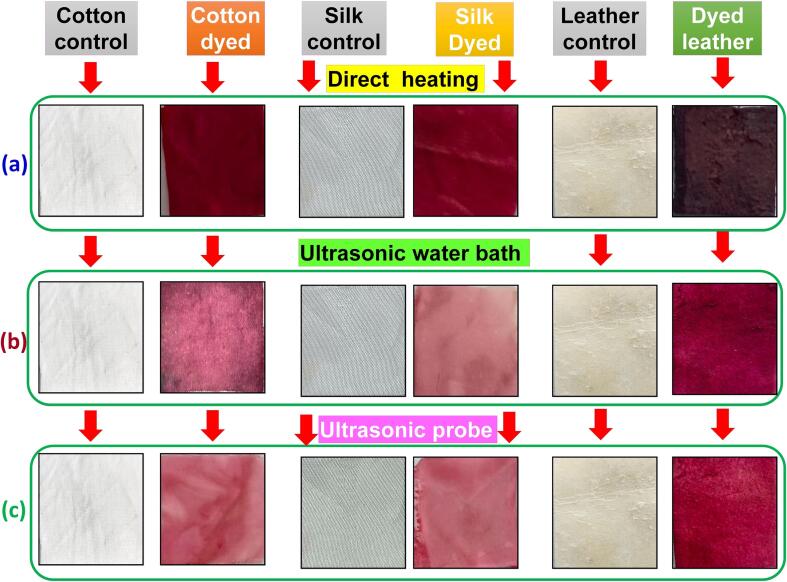
Dyeing of cotton, silk and leather using three different techniques (a) direct heating, (b) ultrasonic water bath and (c) ultrasonic probe with control samples.

**Fig. 6:**
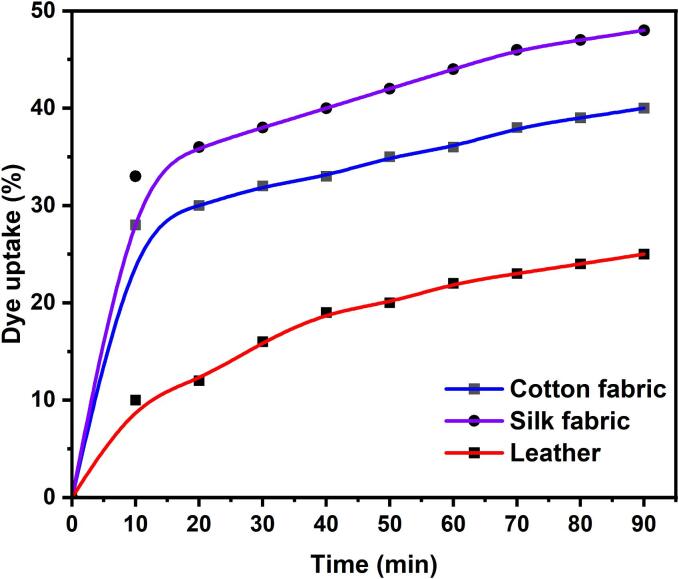
Influence of time on dye uptake during cotton, silk and leather dyeing process under optimized dyeing conditions.

The natural dye extracted from *B. alba* fruit was used for cotton, silk fabric, and leather at 1:30 and 30 °C for different intervals. The dye uptake of cotton, silk fabric, and leather at different dyeing times is presented in Fig. 5, Fig. 6. In the case of cotton and silk fabrics, the dye uptake considerably increased during the initial 30 min of dyeing (Fig. 6), followed by a significant decrease. However, leather required longer dyeing and exhibited lower dye uptake than cotton and silk fabrics (Fig. 5, Fig. 6). The dyeing tipping point was determined to be 30 min. At 30 °C and varying time intervals, cotton, silk fabric, and leather were dyed using *B. alba* fruit red natural dye at a concentration of 6 wt% and a ratio of 1:30. During the initial 30 min of dyeing, the dye uptake showed a significant increase, followed by a notable decrease.

After a prolonged exposure time, dye saturation was observed. Furthermore, the dye uptake was higher than other natural dyes, reaching 45 % after 30 min dyeing [39], [40]. *B. alba* fruit red natural dye was employed to dye cotton, silk fabric, and leather using a liquor-to-dye ratio 1:30.

**Fig. 7:**
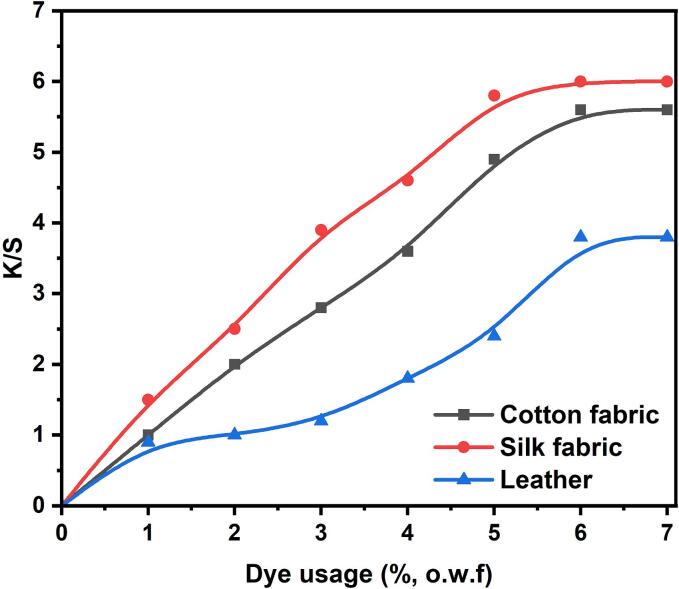
K/S values of the dyed cotton, silk and leather.

The temperature was maintained at 30 °C for 30 min. Fig. 7 depicts the impact of dyes on the K/S values of the dyed leather, silk fabric, and cotton. The K/S value of the coloured silk fabric exhibited a rapid increase as dye usage expanded. However, when the dye consumption exceeded 6 wt%, the growth rate was expected to be more gradual. This indicates that at a dye usage of 6 wt%, the dye absorption had already reached saturation [39], [40].

Practical implications of using B. alba fruit dye in industrial applications

Industrial textiles can use *B. alba* fruit colour. Sustainability is better with *B. alba* fruit dye over synthetic textile colours. Use deep reddish-purple with wool, silk, and cotton to attract eco-conscious shoppers. Biogenic *B. alba* fruit dye reduces textile waste and is safer than synthetic dyes. This encourages ethical and sustainable textile production worldwide. Non-toxic *B. alba* fruit dye may colour food. *B. alba* may replace FD&C Red 40 in beverages, sweets, and processed foods due to demand for safe, plant-based alternatives. Fruit colors may be utilised imaginatively in various dishes, especially acidic beverages containing brilliant crimson anthocyanins, because they are pH-stable. *B. alba* fruit dye makes natural lipsticks, blushes, and eye shadows. Many organic elements make *B. alba* fruit color a skin-friendly alternative to synthetic colors.

### 3.1 Challenges of growth

Optimising Extraction: Lab ultrasonic extraction is promising, but industrial production needs cost, efficiency, and consistency optimisation. Building industrial ultrasonic systems from microscopic probes needs energy, temperature, and solvent management. More research is needed to optimise high-volume extraction parameters without impacting dye quality or yield. Anthocyanins in industrial B. alba fruit colour fade with heat, light, and air. Industry requires encapsulating or stabilising extracted dye. Plant age, soil, and environment impact *B. alba* fruit colour. The challenge demands quality control and consistent extraction. Cosmetics and materials need colour stability. Industrial use of *B. alba* fruit colour involves agricultural and supply chain variables. Sustainable agriculture is needed to meet increased demand for beautiful *B. alba* fruits. Build efficient processing plants near fields to improve agro-industrial collaboration. *B. alba* fruit colouring must be cheaper than synthetics to be popular. By boosting production and optimising the process, natural dye extraction can be cheaper despite its complexity and low yields. Nature's attractiveness may lower synthetic and natural colour prices.

Different solvent extraction protocols were employed to separate natural dyes from *B. alba* fruit. The dyes were then applied to degummed, bleached, and surface-cleaned cotton, silk fabrics, and leather. Fig. 5 and Table 1 present the transparent colour, Integ value and colorimetric parameters of the dyed cotton, silk fabric, and leather under different dyeing methods. Samples 1–4 exhibited transparent colors of yellow–brown, red-brown, brown and dark brown, respectively (Table 2). The Integ value of the samples followed the order 1 < 4 < 3 < 2. During the dyeing process in the direct heating bath, the colour of the dye changed from yellow to brown on cotton fabric, appeared as a light-yellow shade on silk, and transformed into dark brown on leather (Fig. 5). This phenomenon can be attributed to the oxidation of dye molecules occurring in the dye bath, which cannot withstand the direct heating. The dye absorption capacity is also diminished as the heating intensity is reduced. The ultrasonic probe dyeing methods have the drawback of damaging the fabrics and leather when exposed for a long duration, while shorter exposure times result in poor dyeing. However, when the dyeing process is conducted in an ultrasonic bath, where water acts as a barrier to regulate the entry of uniform ultrasound into the liquor bath, it leads to successful dyeing without altering the dye colour (Fig. 5). This ensures uniform adsorption of the dye by the fabric and leather, facilitating the dyeing process without the need for any mordant. Table 2 displays the outcomes of the colour fastness tests conducted on three dyed fabric and leather methods under optimum conditions. The dyed cotton fabric demonstrated good washing and rubbing fastness after two rounds of washing with boiling soap liquid. However, the colour fastness to alkali perspiration received a score of 4 (relatively good) due to the colors sensitivity to pH [41], [42]. During the evaluation, it was observed that the initially dark yellow colour of the dyed leather, silk, and cotton fabric samples became lighter as the pH level increased. This change in pH resulted in a slight reduction in the colour intensity during the alkaline perspiration test.Table 3.

**Table 1:** Color coordination values of dyed cotton, silk fabric, and leather in various dyeing methods at optimum conditions.

Color coordination					
	L*	a*	b*	^△^E	K/S value
**Direct Heating**					
Cotton	33.26	34.67	−1.25	43.28	5.8
Silk	30.32	33.33	−6..52	48.16	7.4
Leather	52.33	18.21	−4.82	28.22	1.0

**Ultrasonic Water bath**					
Cotton	20.16	32.83	−9.35	36.92	5.4
Silk	28.23	34.34	−8.85	38.62	8.2
Leather	46.21	7.35	−2.45	24.21	1.2

**Ultrasonic Probe**					
Cotton	13.18	28.42	−2.36	20.16	4.3
Silk	18.32	36.12	−3.32	22.19	6.9
Leather	51.23	28.52	−10.23	19.24	1.3

**Table 2:** Fastness properties of dyed cotton, silk fabric, and leather in various dyeing methods at optimum conditions.

Ultrasonic Water bath (color cherry red)				
	Rubbing fastness(grade)		Washing fastness (grade)	Light fastness (grade)
	Dry	Wet		
Cotton	4–5	4–5	4	2
Silk	4	4–5	4–5	3
Leather	3	3	3–4	2

**Ultrasonic Probe (color cherry red)**				

	Rubbing fastness (grade)		Washing fastness (grade)	Light fastness (grade)

	Dry	Wet

Cotton	4	4	3–4	2
Silk	3–4	3–4	4	3
Leather	3	3	4–5	2

**Conventional Heating (color cherry red)**				

	Rubbing fastness (grade)		Washing fastness (grade)	Light fastness (grade)

	Dry	Wet

Cotton	4–5	4	4	2
Silk	4	4	3–4	3
Leather	3	3	4	2

**Table 3:** Antibacterial capacity of dyed cotton, silk fabric, and leather.

Bacteria	Undyed cotton fabric	Dyed cotton fabric	BR (%)	Undyed silk fabric	Dyed silk fabric	BR (%)	Undyed leather	Dyed leather	BR (%)
*Staphylococcus* sp.	432	203	53.00	564	124	78.04	850	352	58.58
*Pseudomonas* sp.	526	345	34.41	583	186	68.09	926	364	60.69
*Klebsiella* sp.	468	215	54.05	426	144	66.19	934	645	30.94
*Vibrio* sp.	568	341	39.96	548	187	65.87	998	504	49.49
*Micrococcus* sp.	620	285	54.03	621	206	66.82	942	435	53.82

BR-Bacterial reduction.

**Fig. 8:**
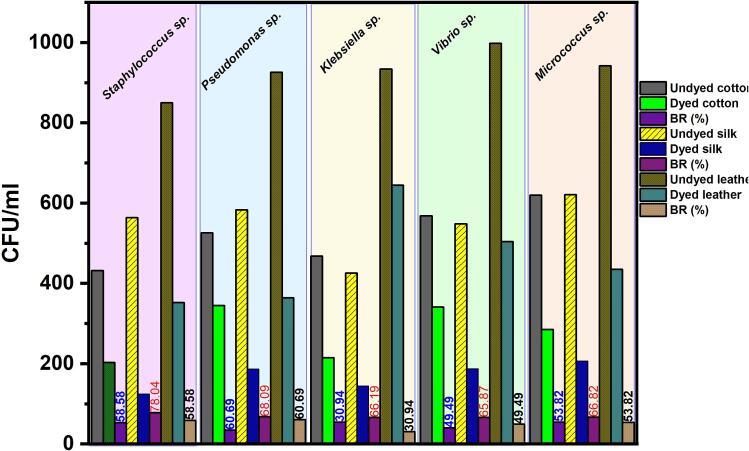
Antibacterial activity of the dyed samples against skin pathogen.

Fig. 8 demonstrates the evaluated antibacterial performance of cotton, silk fabric, and leather. *Staphylococcus* sp, *Pseudomonas* sp, *Vibrio* sp, *Klebsiella* sp, and *Micrococcus* sp exhibited a robust growth in the control medium containing undyed fabrics and leather. In contrast, the presence of dyed cotton, silk fabric and leather in the medium significantly inhibited the growth of *Pseudomonas* sp, leading to a substantial reduction in colony formation by 60.69, 68.09, and 63.23 %, respectively, as shown in Fig. 8. The antibacterial effect can be attributed to the phenolic compounds present in the dye, which can disrupt the enzymes and biofilm structures of bacteria, as well as influence their energy metabolism and selective absorption of chemicals. Notably, the dyed silk materials exhibited exceptional antibacterial properties against *Staphylococcus* sp, *Pseudomonas* sp, *Vibrio* sp, *Klebsiella* sp, and *Micrococcus* sp.

### 3.2 Benefits of excluding mordants in natural dye

Natural dye without mordants offers environmental and health benefits traditional dyeing often uses alum, copper, or chromium mordants. This approach can discharge dangerous compounds into wastewater, harming humans and the environment. By eliminating these compounds, mordant-free dyeing reduces environmental impact and increases worker and consumer safety. Simplified Process: Mordant-free dyeing simplifies dyeing, reduces steps, and makes small-scale, eco-friendly textile production possible. Savings: Large quantities of mordants can be expensive. Avoiding mordants can save money, especially for ecologically conscious businesses and communities.

### 3.3 The challenges of dyeing without mordants

Reduced Colour Fastness: Mordant-free dyeing may fade or wash out faster. This is a major method concern. Since mordants are necessary, colours cannot remain on fibres without them. Restricted Colour Palette: Without mordants, some natural dyes may not attach properly to textile fibres, limiting the quantity and intensity of hues. This may limit the use of some natural dyes in mordant-free methods. Dyeing without a mordant sometimes reduces dye absorption, resulting in less vibrant hues. This may need longer dying times or higher dye concentrations, reducing process efficiency.

### 3.4 Balancing advantages and disadvantages

Recent dye extraction methods including ultrasonic and natural binders are assisting mordant-free dyeing. In addition, plant-based mordants or other ecologically friendly replacements are being studied to improve dye-fiber bonding without compromising environmental benefits.

## 4 Conclusion

Using ultrasound has significantly enhanced the efficiency of extracting red dye from *B. alba* fruit. Specifically, employing 45 W of ultrasonic power for 30 min, it was determined that a 1:1 mixture of ethanol-methanol resulted in higher yields and extraction efficiency. Implementing three distinct extraction techniques has proven beneficial in augmenting the production of the *B. alba* fruit red dye. Among the three extraction protocols, the ultrasonic water bath demonstrated a 6 % increase in dye production when using a 1:1 ethanol-methanol solution with an input power of 45 W. This approach can also be employed for extracting colours from other plant materials, such as *B. alba* fruit, for analytical purposes. Furthermore, the extracted red dye from *B. alba* fruit exhibits excellent colouring capabilities for dyeing cotton, silk, and leather.Moreover, applying ultrasound assistance in natural dyeing processes for cotton, silk, and leather provides benefits, including an improved exhaustion rate. The mechanism behind the improvement observed in ultrasound-assisted extraction could be attributed to the rupturing of the cell walls of flower petals. This rupture facilitates the release and more efficient transport of the *B. alba* fruit red dye into the surrounding medium. This effect is mediated by micro-stirring and acoustic streaming phenomena generated by the cavitation process induced by ultrasound. The current study significantly presents an improved extraction process from natural dye sources, specifically the fruit of *B. alba*, which eliminates the need for external heating. This advancement is particularly beneficial for dyeing cotton, silk, and leather materials. The utilisation of the red dye extracted from the *B. alba* fruit holds potential importance for applications related to sensitive medical issues. Considering the growing environmental concerns, the eco-friendly and non-toxic dyeing of fibre materials, including cotton, silk, and leather, could emerge as a viable “Green chemistry” alternative for dyeing companies soon.

## CRediT authorship contribution statement

**Loganathan Lingeshwaran:** Resources, Project administration. **Jagadeesh Kumar Alagarasan:** Conceptualization. **Seema siddharthan:** Conceptualization. **Kanagasabapathy Sivasubramanian:** Formal analysis. **Palanivel Velmurugan:** Writing – review & editing, Writing – original draft. **Fatimah Oleyan Al-Otibi:** Software, Funding acquisition. **Sivakumar Manickam:** Visualization, Validation, Resources. **Moonyong Lee:** Project administration, Investigation.

## Declaration of competing interest

The authors declare that they have no known competing financial interests or personal relationships that could have appeared to influence the work reported in this paper.
